# ADDRESSING UNMET MENTAL HEALTH NEEDS OF OLDER ADULTS IN TURBO, COLOMBIA: A PILOT INTERVENTION

**DOI:** 10.1093/geroni/igad104.3796

**Published:** 2023-12-21

**Authors:** Clarissa Giebel, Gabriel Saldarriaga, Erika Montoya, Mark Gabbay, Maria Isabel Zuluaga

**Affiliations:** University of Liverpool, Liverpool, England, United Kingdom; Universidad de Antioquia, Medellin, Antioquia, Colombia; Universidad de Antioquia, Medellin, Antioquia, Colombia; University of Liverpool, Liverpool, England, United Kingdom; Universidad de Antioquia, Medellin, Antioquia, Colombia

## Abstract

Mental health is often stigmatised in lower- and middle-income countries, with little resources to provide care. Having lived through extreme and stressful live events in Colombia, including armed conflict, and older adults found to lack adequate mental health support, the aim of this pilot intervention is to improve their mental health. Based on a meta-analysis of community-based mental health interventions for older adults in LMICs, qualitative interviews with older adults and local stakeholders, and a mental health needs assessment survey of the older population in Turbo, Colombia, we consulted older adults in the region to co-produce a community-based intervention. The pilot intervention is running for 12 weeks from May to August 2023, with two sessions provided per week in a community centre. The intervention involves four components - social engagement, educational interventions, physical activities (singing, dancing, cooking), and peer support. We will use a mixed-methods approach to evaluate the effects of the intervention on older adults’ well-being, social support, and loneliness, involving pre- and post-questionnaires and semi-structured interviews. Thirty-five older adults are actively participating in the intervention. Full results will be available in October. This co-produced and evidence-based intervention for older Colombians has the potential to circumvent heavy healthcare resource costs for providing mental health support, and instead involve the community in providing care and support, with trained facilitation. In light of limited mental health support across LMICs, this intervention, if effective, could provide mental health support for older adults in other countries via cultural adaptation.

